# Cytogenetic Abnormalities in Multiple Myeloma: Incidence, Prognostic Significance, and Geographic Heterogeneity in Indian and Western Populations

**DOI:** 10.1159/000529191

**Published:** 2023-02-13

**Authors:** Pratibha Kadam Amare, Shraddha Nikalje Khasnis, Pranita Hande, Hrushikesh Lele, Nishigandha Wable, Snehal Kaskar, Nikita Nikam Gujar, Nilesh Gardi, Aniket Prabhudesai, Karishma Todi, Rohit Waghole, Pritha Roy

**Affiliations:** ^a^Oncocytogenetics and Oncomolecular Department, Lilac Insights Pvt. Ltd, Navi Mumbai, India; ^b^ACTREC, Tata Memorial Center, Navi Mumbai, India

**Keywords:** Multiple myeloma, Cytogenetics, FISH, Geographic heterogeneity

## Abstract

Multiple myeloma (MM) is a genetically complex and heterogeneous neoplasm in which cytogenetics is a major factor playing an important role in the risk stratification of disease. High-risk MM based upon cytogenetic classification includes primary *IGH* translocations t(4;14), t(14;16), t(14;20), and secondary progressive aberrations such as gain/amp(1q), 1p deletion, del(17p), and hypodiploidy. Several studies have proved that interphase FISH can detect primary as well as secondary cryptic aberrations very efficiently in lowest 5–10% abnormal plasma cell population. The present large-scale study was undertaken to evaluate the incidence of cytogenetic abnormalities, to analyse the correlation of conventional karyotyping with FISH, and to seek the geographic heterogeneity in the incidence of primary as well as secondary aberrations in our Indian versus Western populations. We conducted prospective studies of 1,104 patients consecutively referred from the primary, secondary, and tertiary oncology centres from all over India. Interphase FISH was performed on isolated plasma cells. Karyotype analysis was done as per ISCN 2016 and 2020. FISH could detect cytogenetic abnormalities in 67.6% of the cases with an incidence of 59% non-hyperdiploidy. The incidence of *IGH* translocation was 26% versus literature frequency of 40–50% which was mainly due to a low incidence (6%) of t(11;14) in contrast to 15–20% in other series. Additionally, the association of secondary progressive aberrations in the hyperdiploid group rather than the non-hyperdiploid group in our patients is not a common finding. A biallelic inactivation of *TP53* as an ultra-high risk factor was detected in old-aged patients. These observations disclose the novel findings and strongly indicate the racial disparity which leads to geographic heterogeneity. In contrast to FISH, conventional karyotyping could detect MM-related aberrations in 50% of cases, of which 44% revealed highly complex karyotypes with common aberrations of chromosome 1q. Overall, FISH was found to be a novel, easy approach with high success rate and capability of detection of all cytogenetic abnormalities that add valid information for the risk stratification of disease. This, in future, in combination with mutation profile and gene expression profile will help in further refinement of disease and identification of actionable targets.

## Introduction

Multiple myeloma (MM) is a genetically complex and heterogeneous neoplasm in which cytogenetic abnormalities are major genetic factors in the prognostication of disease; hence cytogenetics is considered as an integral part of disease management [Fonesca et al., 2004; Avet-Loiseau et al., 2007; Sawyer, 2011; Rajan and Rajkumar, 2015; Kadam Amare et al., 2016; Sonneveld et al., 2016; Abdallah et al., 2020]. The identification of high-risk and low-risk cytogenetic abnormalities either single or in group plays an important role in the therapeutic decision [Kumar and Rajkumar, 2018; Abdallah et al., 2020; Hanamura, 2022]. Hence, cytogenetics is included in the consensus statement of the European Myeloma Network and International Myeloma Working Group as well as treatment guidelines of the National Comprehensive Cancer Network [Avet-Loiseau et al., 2007; Fonseca et al., 2009].

Trisomies of 3, 5, 7, 9, 11, and 15 are the most commonly observed trisomies in hyperdiploidy which is one of the major cytogenetic groups and is generally considered as low-risk cytogenetic category in MM [Wuilleme et al., 2005; Barilàet al., 2020].

High-risk MM is defined as having at least one of the cytogenetic abnormalities related with poor prognosis, which include *IGH* translocations t(4;14), t(14;16), and t(14;20), del (17p), p53 mutation, 1q gain/amplification, and 1p deletion. The genes dysregulated in the high-risk translocations are: 4p16.3 (*FGFR3* and *MMSET*), 16q23 (*c-MAF*), and 20q11 (*MAFB*) [Bergsagel et al., 2013; Gozzetti et al., 2014; Sonneveld et al., 2016].

Recently, the Mayo Clinic has proposed a concept similar to high-grade lymphomas which is described as double-hit MM, having two high-risk, and triple-hit MM, with three high-risk abnormalities which seem to be additional adduct, helpful prognostic indicators in the understanding of the MM prognosis [Walker et al., 2019; Abdallah et al., 2020].

Biallelic inactivation of *TP53*, occurring in 2–4% of newly diagnosed MM patients, was identified as an ultra-high-risk feature of MM, being associated with a median survival of less than 2 years [Ashby et al., 2019; Munawar et al., 2019].

In comparison to conventional metaphase cytogenetics which yields a poor mitotic index, unable to give a true picture of cytogenetics, several groups have proved that interphase FISH can efficiently detect more than 90% of cytogenetic abnormalities including cryptic 13q deletion, various *IGH* translocations, aberrations of 17p, 1q gain/amplification, 1p deletion, and trisomies of odd numbered chromosomes in 60–90% of MM patients [Avet-Loiseau et al., 2007; Fonesca et al., 2009; Rajan and Rajkumar, 2015; Kadam Amare et al., 2016; Kumar and Rajkumar, 2018; Abdallah et al., 2020].

The present large-scale study was undertaken to (1) evaluate the incidence of cytogenetic abnormalities, (2) assess the frequency of double-hit and triple-hit myeloma, (3) analyse the correlation of metaphase cytogenetics with FISH analysis, and, most importantly, (4) seek the geographic heterogeneity in the incidence of the abnormalities in Indian and western countries' populations.

## Materials and Methods

### Patients

We conducted prospective studies on 1,104 consecutive patients (675 males, 429 females, age range 30–96 years) who were newly diagnosed MM cases from all the zones of Indian population which included the tertiary oncology hospitals, polyclinics, individual clinics, since January 2016 to December 2022. The diagnosis of MM was evaluated and confirmed by bone marrow pathology and immunobiochemical parameters. The exclusion criteria were history of other primary malignancies, administration of chemotherapy or radiotherapy before diagnosis of MM.

Plasma cell isolation was performed by purification of plasma cells using CD138-coated magnetic beads according to the manufacturer's instructions (Miltenyi Biotec, Paris, France).

### FISH on Plasma Cells

Mononuclear cells from bone marrow aspirate were enriched by Ficoll Hypaque gradient centrifugation. Plasma cells were purified by using CD138-coated magnetic beads according to the manufacturer's instructions. Enriched plasma cells were identified by FITC-conjugated anti-human Kappa Lambda Light Chain staining and the purity was 95% (range 70–99%).

Interphase FISH was performed on isolated plasma cells using locus-specific probes SPEC *RB1*/13q12 dual colour probe/LSI 13q34 (control), SPEC *TP53* (17p13)/CEN17 dual colour probe, SPEC *CKS1B*, *CDKN2C* dual colour, LSI break apart dual colour 5′-3′ *IGH* probe, dual fusion *IGH* translocation probes *CCND1/IGH*, *MAF/IGH*, *IGH/MAFB*, *MYC 5*′*-3*′ break apart probe (ZytoLight Spec, Germany), Metasystem XL *IGH/FGFR3 DF*, *CCND3/IGH* probe. Hyperdiploidy was analysed by using a set of probes specific for CEN3, CEN7 (ZytoLight), and XL5p15/9q22/15q22 (Metasystems). Hyperdiploid MM was defined as presence of trisomy of ≥2 odd numbered chromosomes [Ashby et al., 2019; Abdallah et al., 2020]. FISH procedure was followed as per manufacturer's protocol. A total of 200 interphase plasma cell nuclei were evaluated by two observers. The cut off threshold for ∆13 (del(13q)/–13), del(17)(p13.1), 1q gain/amp was 5%, t(14q32) was 10%, for dual fusion translocation probes (*CCND1/IGH*, *FGFR3/IGH*, *MAF/IGH*, *MAFB/IGH*, *CCND3/IGH*), *MYC* (5′-3′ *MYC* break-apart) and trisomy was 5%. Results were considered abnormal if the percentage of nuclei with the abnormal hybridization signals was >3 SD from the mean. Two technologists scored the results. For biallelic *TP53* inactivation *TP53* mutation analysis was performed by Sanger sequencing in 48 cases with del(17p).

Bone marrow samples from 30 individuals without apparent haematological diseases and with normal karyotypes were used as controls. Means and SD of the percentages of nuclei with one, two, three, or break apart hybridization signals were obtained and calculated on at least 200 cells.

### Conventional Karyotyping

The karyotypes were obtained by culturing bone marrow aspirate for 4–5 days in complete medium with B-cell mitogens interleukin 4 (250 ng) and CD40L (400 ng). The cultures were harvested by hypotonic KCl treatment followed by methanol:acetic acid fixative, and mitotic preparation were made for GTG staining [Ding et al., 2013]. A minimum of 20 cells were karyotyped and abnormalities were defined as per ISCN 2016 and ISCN 2020 [McGowan-Jordan et al., 2016, 2020]. GTG-banded karyotype analysis was confirmed by WCP on metaphase cells and interphase FISH.

### Statistical Analysis

Results of all 1,104 cases were enrolled for statistical analysis to investigate frequency of cytogenetic abnormalities, association of hyperdiploidy and non-hyperdiploidy with *IGH* translocation and other high-risk markers, incidence of dual- and triple-hit myeloma along with incidence of frequent high-risk markers in dual and triple-hit MM. Fisher's exact test at a significance level of 0.05 was used to compare the groups. All statistical analyses were carried out in R software. A comparison of incidence among different chromosome abnormality groups was analysed using a proportion test.

## Results

### Conventional Karyotyping

**Fig. 1:**
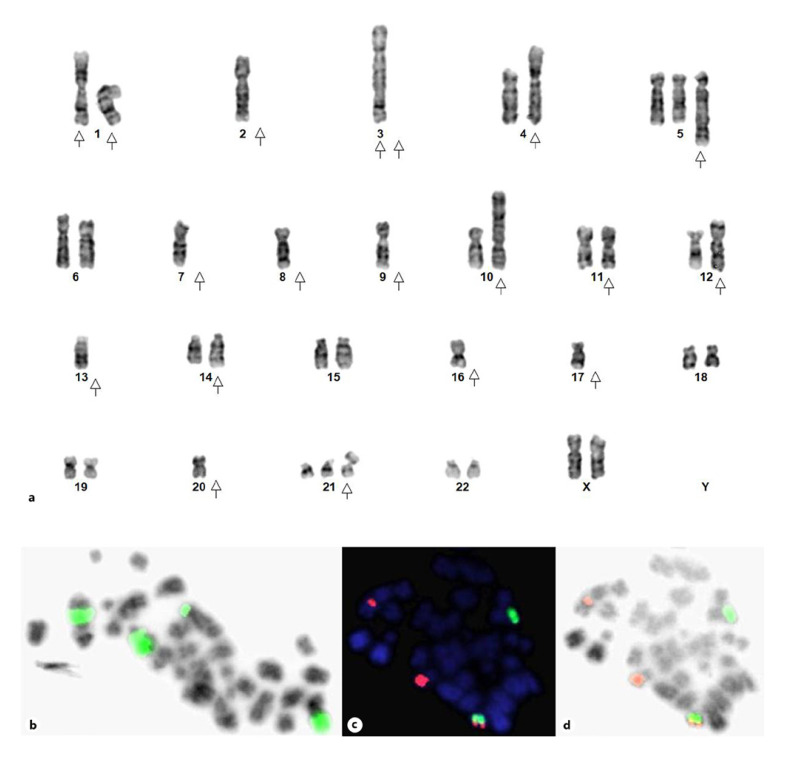
**a** G-banded karyotype. 42,XX,+1der(1)del(1)(p13)dup(1)(p22q42),t(1;3)(q22;p21)t(1;10;3)(p22;p13;q22)t(1;4)(p32;p14),–2,–3,+5?t(5;8)(q31;q22),–7,–8,–9,t(11;?)(p15;?),t(12;17)(p11.2;p11.2),–13,t(14;16)(q32;q23),t(1;16)(?p13;q23),–17,–20,+21i(21)(q10). The complex karyotype was checked and confirmed by FISH with WCP14 and 16 probes. Arrows indicate structural and numerical chromosome abnormalities. **b** Inverted DAPI metaphase image after FISH with WCP1 showing duplications (gain of 1q copy number) followed by translocation and insertion to other chromosomes indicating the mechanism of chromothripsis. **c** WCP14 (green) and WCP16 (red) showing *IGH/MAF* co-localization: t(14;16)(q32;q23). **d** Inverted DAPI image of the same metaphase.

**Fig. 2:**
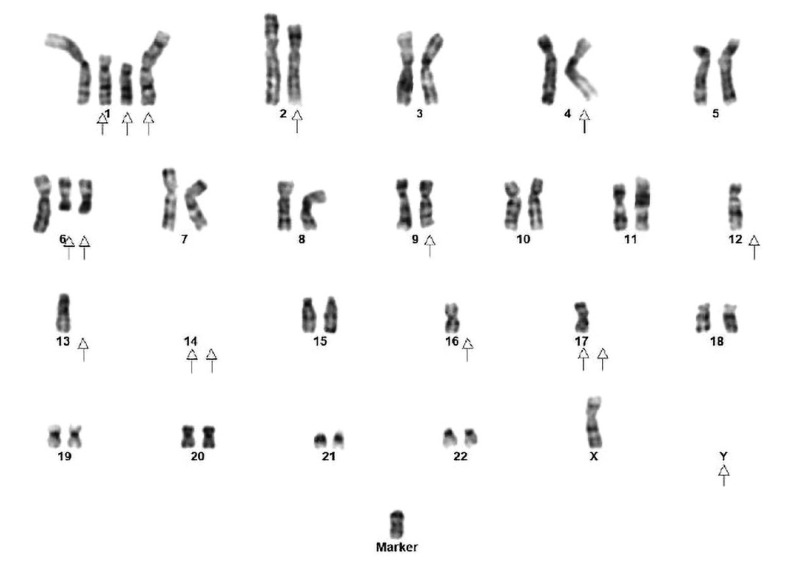
G-banded karyotype. 43,X,–Y,+1,+der(1)t(?;1)(?;p32),del(1)(p13)×2,del(2)(p23),t(4;14;?)(p16;q32;?),+6del(6)(q21)×2,t(?;9)(?;q22),–12,–13,–14,–16,–17,t(?;17)(?;p13),+mar. Arrows indicate structural and numerical chromosome abnormalities.

In conventional cytogenetic preparations, successful results were obtained in 65 of 80 cases (80%). Diploid, hyperdiploid (47–59 chromosmes), hypodiploid (40–45 chromosomes) and triploid-tetraploid (61–90 chromosomes) karyotypes were observed in 13 (20%), 19 (29.2%), 12 (18.4%), and 4 (6%) cases, respectively. Abnormalities of inv(9), inv(Y) or loss of Y were common findings in 16 cases with hypodiploid and diploid karyotypes. MM FISH panel was negative in 32 of 65 (50%) cases. Twenty-one out of 65 (32.3%) cases were either hyperdiploid or had 1–2 non-recurrent abnormalities which were not related to MM. Overall, 29 of 65 cases (44.6%) had highly complex karyotypes with abnormalities of chromosome 1 and del(17)(p11.2). Abnormalities of chromosome 1 included homogeneously staining regions (HSR) and multiple events of chromosome 1 such as deletion of 1p, duplication of 1q, 1q translocation, and insertion into other chromosomes at random (Fig. 1, 2). Overall 17/32 cases (53%), those with 1q gain/amp showed either extra copies of 1 or duplication of 1q through i(1q) or HSR on 1q followed by translocation, insertion at other chromosomes at random (Fig. 1, 2). Hyperdiploid karyotypes revealed gain of almost all chromosomes, of which gain of odd numbered chromosomes like 3, 5, 7, 9, 15, 19, 21 was common. The interesting observation was that all complex karyotypes with chromosome 1 abnormalities revealed gain/amplification of 1q. Hypodiploid karyotypes revealed losses of chromosomes 13, 14, 16, and 20.

### Incidence of Overall Abnormalities, Hyperdiploidy, *IGH* Translocations, and High-Risk Abnormalities by FISH

Among 1,104 cases, 160 cases (14.55%) were in the age range of 30–50 years and 944 cases (85.5%) were between 51 and 96 years. The male:female ratio was 1.57:1.

Of the 1,104 patients, 746 (67.6%) had chromosome abnormalities, which included hyperdiploidy, *IGH* translocations, and high-risk abnormalities. Abnormal clone size was 5–100%.

Finally, 655 (59.2%) cases had non-hyperdiploidy and 40.8% had hyperdiploidy. Hyperdiploidy revealed common gain of odd numbered chromosomes 3, 7, 9, and 15, of which gain of chromosome 3 was 28.4%, chromosome 7: 22.2%, chromosome 9: 33.05%, and chromosome 15: 36.2%. In a separate cohort of 743 cases, chromosome 5 aneuploidy was checked along with chromosomes 3, 7, 9, and 15. Gains of chromosomes 5, 9, 15 were higher, i.e., 33%, 33.6%, and 55%, respectively as compared to gains of chromosomes 3 and 7 (27.6% and 20.4%, respectively). Gain of chromosome 15 was highest (55%) among the gains of 3, 7, 5, 9, and 15.

### Incidence of Chromosome Abnormalities in Our Series

The incidence of chromosome abnormalities among the 1,104 cases was as follows: chromosome 13 abnormalities 384/1,104 (34.7%) which included del(13q): 38/1,104 (3.4%) and monosomy 346/1,104 (31.3%); gain/amp(1q): 357/1,104 (32%), gain(1q): 209/1,104 (19%), amp(1q): 148/1,104 (13%); aberration of chromosome 17: 112/1,104 (10.2%), del(17p): 87/1,104 (8%), −17: 15/1,104 (1.3%), dup(17p): 10/1,104 (0.9%); del(1p): 63/1,104 (5.7%); *IGH* translocations: 289/1,104 (26%) and *IGH* partial deletion 82/1,104 (7.4%) (Table 1).

**Fig. 3:**
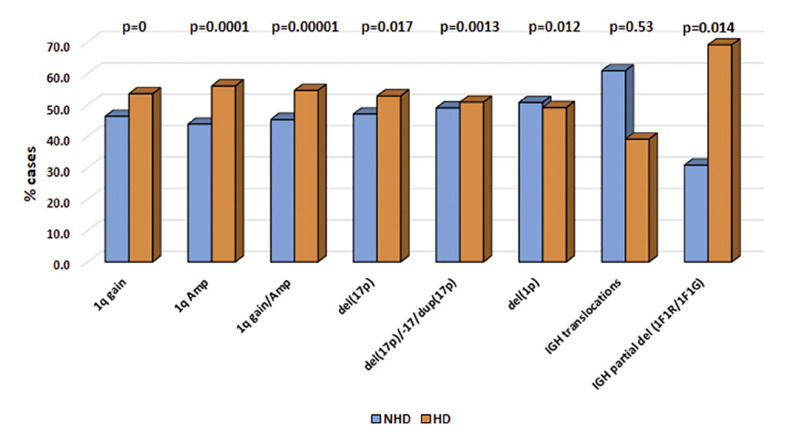
High-risk markers in the non-hyperdiploid (NHD) and hyperdiploid group (HD).

Clustering of abnormalities viz, del(13q), −13, gain/amp(1q), del(17p), −17, dup(17p), del(1p), *IGH* translocations, *IGH* partial deletion, was detected in both the non-hyperdiploid as well as in the hyperdiploid group. There was no significant association of high-risk markers with the non-hyperdiploid group. It was noted that aberration of 17 (*p* ≤ 0.00135) and gain/amp(1q) (*p* = 0.00001) and *IGH* partial deletion (*p* < 0.014) were associated with the hyperdiploid group (Fig. 3).

**Fig. 4:**
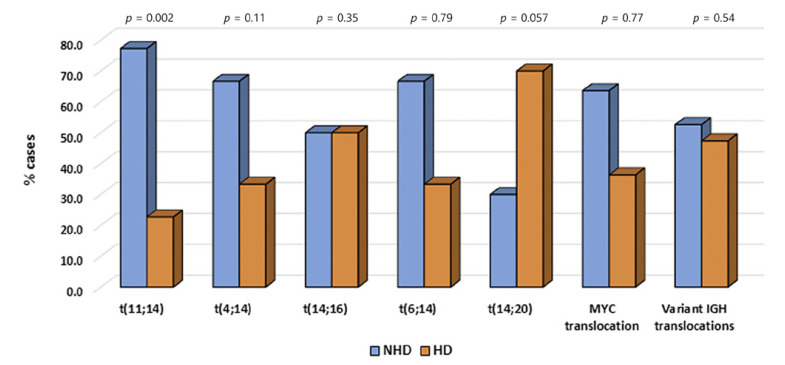
*IGH* translocations in the non-hyperdiploid (NHD) and hyperdiploid group (HD).

Recurrent *IGH* translocations were detected as follows: t(11;14): 66/1,104 (6%), t(4;14): 99/1,104 (9%), t(14;16): 24/1,104 (2.1%), t(6;14): 3/1,104 (0.27%), t(14;20): 10/1,104 (0.9%), *MYC* translocations: 11/1,104 (1%), and variant *IGH* translocations: 19/1,104 (1.7%) (Fig. 4).

Although *IGH* translocations including t(4;14) were more frequent in non-hyperdiploid group than in the hyperdiploid group, there was no significant association of t(4;14), t(14;16), t(6;14), t(14;20), *MYC* translocations, variant *IGH* translocations with the non-hyperdiploid group except t(11;14) which was more prevalent in the non-hyperdiploid group (*p* ≤ 0.002) (Fig. 4).

### Association of High-Risk Markers and Monosomy 13 with *IGH* Translocations

Clustering of monosomy 13 was detected in t(4;14)-positive cases (*p* ≤ 0.00001). The del(17p)/–17/dup(17p) abnormalities were associated with t(11;14) (*p* ≤ 0.0178), t(4;14) (*p* ≤ 0.00001), t(14;16) (*p* ≤ 0.0005), and variant *IGH* translocations (*p* ≤ 0.00001). The significant association of gain/amp (1q) was seen in t(4;14) (*p* ≤ 0.00001), t(14;16) (*p* ≤ 0.000017), t(14;20) (*p* ≤ 0.016), *MYC* translocations (*p* ≤ 0.001), and variant *IGH* translocation (*p* ≤ 0.025) positive cases. The del(1p) occurred frequently in t(14;16) (*p* ≤ 0.043) positive cases.

### Characteristics of Double- and Triple-Hit Myeloma

**Fig. 5:**
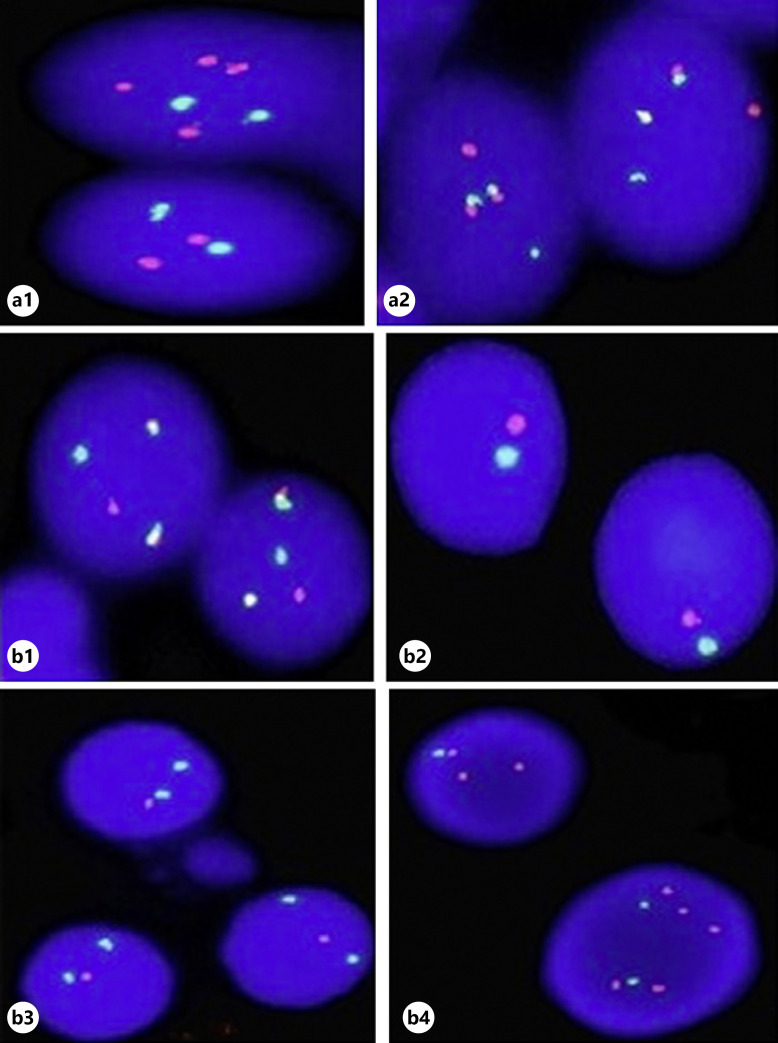
FISH on interphase cells. **a** Double-hit myeloma. **a1** 1q amplification; **a2** *IGH/MAFB* colocalization: t(14;20)(q32;q12). **b** Triple-hit myeloma. **b1** *IGH/FGFR3* colocalization: t(4;14)(p16;q32); **b2** monosomy of chromosome 13; **b3** 17p deletion; **b4** 1q amplification as well as 1p deletion.

The incidence of double-hit MM was 13.3% (147/1,104 cases) and triple-hit MM was 5% (55/1,104 cases). We did not find significant association of double- and triple-hit MM with ploidy group (double-hit with hyperdiploidy, *p* ≤ 0.096; triple-hit with hyperdiploidy, *p* ≤ 0.3; double hit with non-hyperdiploidy, *p* ≤ 0.096; and triple-hit with non-hyperdiploidy, *p* ≤ 0.306). Further, there was no prevalence of double- and triple-hit MM in old aged cases [≤60 years, *p* < 0.92 vs. ≥60 years, *p* ≤ 0.74) (Fig. 5a, b).

Double-hit MM was more prevalent in t(4;14) (*p* ≤ 0.00001), t(14;16) (*p* ≤ 0.0001), t(14;20) (*p* ≤ 0.0007), *MYC* translocations (*p* ≤ 0.0013), and variant *IGH* translocations (*p* ≤ 0.05). Similarly, triple-hit MM occurred frequently in t(4;14) (*p* ≤ 0.00001), t(14;16) (*p* ≤ 0.00001), and *MYC* translocations (*p* ≤ 0.014).

All high-risk abnormalities such as abnormalities of del(17p)/–17, gain/amp(1q), and del(1p) were most common in double-hit MM (*p* ≤ 0.000036, *p* ≤ 0.00001, and *p* ≤ 0.00001, respectively) and triple-hit MM (*p* ≤ 0.00001, *p* ≤ 0.00001, *p* ≤ 0.00001, respectively). Monosomy 13 was also frequently detected in double-hit (*p* ≤ 0.00001) and triple-hit MM (*p* ≤ 0.0024) (Fig. 5a, b) There was no age-related prevalence of high-risk abnormalities as the frequencies of single-hit, double-hit, and triple-hit MM were almost the same in cases ≤60 years and ≥60 years (*p* ≤ 0.363, *p* ≤ 0.92, *p* ≤ 0.74, respectively).

While screening for the *IGH* translocation, *IGH* partial deletion was noted in all *IGH* translocation-positive cases with frequency 2.3–39%. The association of *IGH* partial deletion was more common in t(4;14) (39%).

A biallelic *TP53* mutation study in 48 cases with del(17p) revealed 7 cases (14.6%) with a biallelic *TP53* mutation. Out of 7 cases, 6 were above 60 years, indicating total inactivation of *TP53* is a very high risk factor that could emerge during the ageing process, which might cause further progression of the disease.

## Discussion

### Fluorescence in situ Hybridization

In the present study of 1,104 cases, sensitive FISH on purified plasma cells could detect genomic abnormalities in 67.6% of cases which was almost comparable to the incidence (50–90%) reported in literature [Avet-Loiseau et al., 2007; Jacobus et al., 2011; Rajan and Rajkumar, 2015; Sonneveld et al., 2016]. The incidence of higher frequencies of chromosomes 5, 9, and 15 than chromosomes 3 and 7 in hyperdiploidy is in agreement with reported studies [Kumar et al., 2012]. Losses of chromosomes 13, 14, 16 observed in hypodiploid karyotypes by conventional karyotyping are generally common losses in hypodiploid MM [Smadja et al., 2001; Van Wier et al., 2013]. The frequencies of del(13q), monosomy 13, gain/amp(1q), del(17p) observed in our cohort do not differ markedly from those reported by others, except frequency of del(1p) was comparatively low (5.7%) as compared with 15–30% reported frequency [Hebraud et al., 2014; Walker et al., 2015].

*IGH* partial deletion studies are scarce in the literature. The present study found that the low frequency (7.4%) of *IGH* partial deletion either telomeric 5′ or 3′ centromeric is due to an *IGH* fusion most frequently in t(11;14), t(4;14), and t(14;16) [Rajkumar, 2020; Smith et al., 2020]. As reported by Rajkumar [2020], we also found that the majority of these *IGH* partial deletions were more common in t(11;14), t(4;14), and t(14;16) cases which are generally associated with worse outcome except t(11;14) [Rajkumar, 2020; Smith et al., 2020].

The incidence of overall *IGH* translocations (26%) is lower than the reported frequencies of 40–50% [Fonesca et al., 2004; Avet-Loiseau et al., 2007; Schmidt-Hieber et al., 2012; Rajan and Rajkumar, 2015; Kumar and Rajkumar, 2018]. The main reason for lower *IGH* translocation frequency is due to very low frequency of t(11;14) (6%) as compared to other series (15–20%). Greenberg et al. [2015] reported significantly lower frequency of t(11;14) in blacks compared with whites. They also found low frequency of t(4;14) in blacks as compared with whites [Fonesca et al., 2004, 2009; Greenberg et al., 2015]. Our previous study has also reported the low frequency of t(11;14) in Indian population [Kadam Amare et al., 2016].

The association of high-risk markers, i.e., aberration of chromosome 17 [del(17p)/–17/dup(17p)], only del(17p), gain/amp(1q), and *IGH* partial deletion with the hyperdiploid group rather than the non-hyperdiploid group observed in our large cohort has not been reported by previous studies [Fonesca et al., 2004; Sawyer; 2011; Barilà et al., 2020], which further supports and strongly indicates the racial disparity which leads to geographic heterogeneity.

As reported in published studies, various *IGH* translocations preferably occurred in the non-hyperdiploid group. However, our study highlights that only t(11;14) showed significant association with the non-hyperdiploid group (Fig. 4) [Avet-Loiseau et al., 2002, 2007; Sonneveld et al., 2016]. Further, our study revealed that the frequency of occurrence of high-risk markers like aberration of 17p, gain/amp(1q), including monosomy 13, was associated with t(11;14), t(4;14), t(14;16), t(14;20), and variant *IGH* translocations, which further supports the concept of primary *IGH* translocation which later on develops high-risk abnormalities as secondary, progressive events. *MYC* was associated only with gain/amp(1q), which suggests that *MYC* translocations are themselves secondary rather than primary changes [Avet-Loiseau et al., 2007; Walker et al., 2015; Weinhold et al., 2016].

The frequent occurrence of t(4;14), t(14;16), t(14;20), *MYC* translocations in double-hit MM, and t(4;14), t(14;16), and *MYC* translocations in triple-hit MM indicates that t(14;16), t(14;20), and *MYC* translocations are high-risk translocations.

There is a controversy whether monosomy 13 can be considered as a high-risk abnormality [Rajan and Rajkumar, 2015; Rajkumar, 2015; Binder et al., 2017; Kumar and Rajkumar, 2018]. The strong association of monosomy 13 in double- and triple-hit MM like the association of other high-risk abnormalities such as aberrations of 17p, gain/amp(1q), and del(1p), supports the notion that monosomy 13 can also be considered as a high-risk abnormality. A biallelic inactivation of *TP53* as an ultra-high risk factor was detected more in cases above 60 years, indicating progressive changes in old-aged patients.

Gain/amp(1q) leads to gain/amplification of several genes such as *ANP32E*, *MCL1*, *PSMD4*, *ILF2*, *IL6R*, and *PBX1* apart from *CKS1B* which leads to overexpression of these genes which may affect the resistance to different drugs [Sawyer et al., 2005; Shaughnessy, 2005; Hanamura et al., 2006; Shah et al., 2017; Zeng et al., 2019; Hanamura, 2022]. Similarly, FISH studies in MM show deletion of *CDKN2C* on 1p. There are several deletion loci on 1p arm. The candidate genes on del(1p) are *CDKN2C* and *FAF1* at 1p32, *RRLP* and *EVI5* at 1p22 and *FAM46C* at 1p12. *CDKN2C* performs an important function in cell cycle inhibition, hence 1p deletion plays an important role in cell proliferation in MM [Hebraud et al., 2014; Barbieri et al., 2016; Walker et al., 2019]. Whole-genome screening by oligo-based microarrays and aCGH technique have identified CNAs >100 kb in 100% of cases. Most common CNAs were found in 1p, 1q, 6p, 8p, 13q, 14q, 16q, and 22q along with gain of extra copies of odd-numbered chromosomes [Smetana et al., 2014].

### Conventional Karyotyping

Conventional karyotyping studies in MM have been able to detect only 20–30% cells with abnormal karyotypes due to low proliferative index of G0 plasma cells in which cryptic translocations/insertions like t(4;14), t(14;16) were missed [Sawyer et al., 1998; Chang et al., 2004]. On the other hand, karyotyping gives a global view of most of the known and unknown aberrations [Sawyer et al., 1998, 2014; Chang et al., 2004; Soekojo et al., 2019]. We could detect copy number changes in 1q along with translocation/insertion to multiple chromosomes, which cannot be detected by FISH. The amplification of 1q seems to be the result of pericentromeric instability which results in increase in the 1q copy number followed by translocation, insertion, inverted duplication randomly to various chromosomes by jumping rearrangements (Fig. 1a, b). The mechanism is called chromothripsis, which involves chromosomal shattering and random reassembly and localized clustering of breakpoints in response to one catastrophic event rather than accumulation of a series of subsequent events. This results in copy number changes of 1q genes, described as gain/amp(1q) here, which can be very well studied by application of FISH [Cai et al., 2014; Morishita et al., 2016; Le Baccon et al., 2001; Maura et al., 2022]. There is also a chance of missing cryptic translocations like t(4;14)(p16;q32) and t(14;16)(q32;q23) at chromosome level. The karyotyping revealed hypodiploidy with losses of chromosomes 13, 14, and 16 which one cannot detect by FISH studies, and detection of hypodiploidy in MM is very important because it is associated with very poor prognosis [Van Wier et al., 2013; Sawyer et al., 2017]. There are always some advantages and disadvantages with either FISH or conventional karyotyping. However, considering the easy approach, the success rate even at an early stage and expected important valid information from the prognostic, therapeutic point of view, FISH seems to be a gold standard technique to study the genomic picture in MM.

In conclusion, the present study represents the first large-scale evaluation of cytogenetic abnormalities in MM in an Indian cohort by conventional karyotyping complemented by FISH.

FISH could detect 59% of non-hyperdiploidy which was higher than the incidence reported in the literature. The association of secondary abnormalities with all *IGH* translocations supports the concept that primary *IGH* translocations later on develop high-risk abnormalities as secondary progressive events. The strong association of monosomy 13 like other high-risk abnormalities in double- and triple-hit MM indicate that monosomy 13 can also be considered as a high-risk abnormality, which is still a dilemma.

The incidence of *IGH* translocations was 26% versus a literature frequency of 40–50%. The said frequency was mainly affected by low occurrence of t(11;14) (6%) in contrast to 15–20% in other studies. Additionally, the association of high-risk markers such as abnormalities of chromosome 17, gain/amp(1q), and *IGH* partial deletion in the hyperdiploid group rather than non-hyperdiploid group in our patients is not a common finding. These findings support and strongly indicate the racial disparity which leads to geographic heterogeneity. As reported by many other studies, the conventional karyotyping could not reveal the true picture of MM cytogenetics in all cases. The detection of 1q copy number changes along with translocation, insertion to multiple chromosomes, also hypodiploidy with losses of chromosomes 13, 14, and 16 cannot be detected by interphase FISH. However, due to its overall easy approach, success rate, capability of identifying cryptic aberrations that add valid information from the prognostic, risk stratification, and therapeutic decision point of view, FISH seems to be the gold standard, indispensable technique to study the cytogenomic status in MM. Further, in future, prognostication in MM needs involvement of an international staging system, cytogenetics, mutation profile in combination with genome expression profile which will help refinement of prognostication and implementation of genome-guided targeted therapies.

## Statement of Ethics

All experimental procedures were approved by the ethics committee of the Lilac insights Pvt Ltd. headed by Mr. Subhamoy Dastidar (Director), Dr. Pratibha Kadam Amare (Chief and Lab Director), Dr. Chaitanya Datar (Consultant), Dr. Balkrishna Padate (Consultant), Reference No. LIPL/EC/001. As the study was conducted on specimens received in a lab for diagnosis purpose, no clinical trial was involved. Support regarding specimen consent was given by clinicians mentioned in the acknowledgement. Written informed consent was obtained from participants prior to the study.

## Conflict of Interest Statement

All the authors have no conflict of interest to disclose.

## Funding Sources

This study was funded by Lilac Insights Pvt. Ltd.

## Author Contributions

P. Kadam Amare: Conceived and designed the experiments and wrote the manuscript. S. Nikalje Khasnis, P. Hande, H. Lele, N. Wable, S. Kaskar, N. Nikam Gujar, A. Prabhudesai, K. Todi, R. Waghole, and P. Roy: Performed the experiments and analysed the data. N. Gardi: Performed statistical analysis.

## Data Availability Statement

The data corresponding to the above research article are present with Lilac insights Pvt. Ltd.

## Figures and Tables

**Table 1:** Incidence of chromosome abnormalities in 1,104 multiple myeloma cases

Chromosome abnormality	Frequency, %
del(13q)	3.4
Monosomy 13	31.3
Gain(1q)	19
amp(1q)	13
Gain/amp(1q)	32
del(17p)	8
Monosomy 17	1.3
dup(17p)	0.9
del(1p)	5.7
*IGH* translocations	26
*IGH* partial deletion	7.4
